# Testosterona and suicide

**DOI:** 10.1192/j.eurpsy.2022.2181

**Published:** 2022-09-01

**Authors:** L. Gallardo Borge, I.D.L.M. Santos Carrasco, P. Marqués Cabezas, A.I. Segura Rodríguez, G. Medina Ojeda

**Affiliations:** 1 Hospital San Pedro, Psychiatry, Logroño, Spain; 2 Hospital Clínico Universitario, Psychiatry, Valladolid, Spain

**Keywords:** Suicide, Impulsivity, Transgender, Testosterone

## Abstract

**Introduction:**

Testosterone is an anabolic androgenic steroid hormone involved in brain development, reproduction, and social behavior. Several studies have shown that testosterone can cause impulsivity in humans. This impulsivity could modify the mood and increase the risk of suicidal behaviour.

**Objectives:**

Testosterone is an anabolic androgenic steroid hormone involved in brain development, reproduction, and social behavior. Several studies have shown that testosterone can cause impulsivity in humans. This impulsivity could modify the mood and increase the risk of suicidal behaviour.

**Methods:**

Clinical case and literature review.

**Results:**

A 33-years male (biological female), single, gypsy ethnicity, with an 11-years daughter. Psychiatric history of one admission in a hospitalization unit. Diagnosed of depressive disorder and personality disorder NOS. Intermittent follow-up in Mental Health consultations. 8 years later, he consulted due to gender dysphoria. He refered not to be feeling identified with his body for a long time. He rejected his sexual characteristics. After his mental evaluation, he was refered to Endocrinology Service. He had been prescribed with testosterone. Three days after starting the treatment, he maked anattempt of suicide with medication. The patient had not presented previous suicide attempts or ideation. With the withdrawal of the testosterone, the suicidal behaviour dissapeared.

**Conclusions:**

Due to the association of testosterone and suicidal behavoiur, we consider that is important to pay attention to people who have just started the androgenic treatment in order to avoid a high risk of suicide. In the same way, we should focus on evaluating the hostility, impulsivity and irritability in patients strongly related to suicidal behaviour.

**Disclosure:**

No significant relationships.

